# Differential ion selectivity and disease-associated dysfunction of TRPML channels revealed by patient and engineered mutants

**DOI:** 10.1016/j.jbc.2025.110953

**Published:** 2025-11-17

**Authors:** Braden E. Rue, Anna M. Dischler, Lyndsie A. Salvagio, Michael Zhu, Gabriel Xu, Patrick C. Flores, Chelsea L. Donovan, Xin Liu, Taylor F. Minckley, Brooke Agulnek, Yan Qin

**Affiliations:** 1 Department of Biological Sciences, University of Denver, Denver, Colorado, USA; 2 Cherry Creek High School, Greenwood Village, Colorado, USA

**Keywords:** transient receptor potential channels, mucolipidosis type IV, calcium, zinc, permeability, lysosome

## Abstract

The endolysosomal transient receptor potential mucolipin (TRPML) channels play key roles in regulating lysosomal trafficking, signaling, and function. While mutations in TRPML1 cause mucolipidosis type IV (MLIV), the functional consequences of many disease-associated mutations remain unclear. In this study, we used live-cell confocal imaging in HeLa cells to comprehensively characterize the subcellular localization and cation (Ca^2+^ and Zn^2+^) permeability of TRPML1-3, 10 TRPML1 MLIV patient–derived mutants, and engineered pore mutants. We showed that TRPML1 and TRPML3 permeate both Ca^2+^ and Zn^2+^, whereas TRPML2 conducts only Ca^2+^. Subcellular localization analyses revealed that TRPML1 and TRPML2 localize predominantly to lysosomes, whereas TRPML3 is preferentially localized to the endoplasmic reticulum. Among the 10 patient-derived TRPML1 mutants, nine exhibited severely impaired agonist-mediated Ca^2+^ and Zn^2+^ permeability, indicating severe functional loss. In contrast, the F408Δ mutant, associated with a milder phenotype, retained partial ion permeability and was the only mutant capable of constitutive Ca^2+^ permeation without agonist stimulation. Interestingly, we found that nonfunctional, lysosome-localized TRPML1 mutants are associated with more severe disease phenotypes than those retained in the endoplasmic reticulum, suggesting that lysosomal localization of nonfunctional TRPML1 may have dominant-negative or toxic effects. Finally, through structure-guided mutagenesis, we generated a metal-selective TRPML1 mutant, I468V, that is permeable to Ca^2+^ but not to Zn^2+^, providing a new tool for dissecting the distinct physiological roles of Zn^2+^ and Ca^2+^ in TRPML1-mediated processes. Together, these findings provide new insights into how TRPML1 mutations disrupt subcellular localization, ion permeability, and selectivity, which contribute to the variable clinical spectrum of MLIV.

Transient receptor potential mucolipin 1 (TRPML1) is a nonselective cation channel localized to the membranes of late endosomes and lysosomes and is permeable to Ca^2+^, Fe^2+^, Mn^2+^, and Zn^2+^ (1, 2, 3). TRPML1 is ubiquitously expressed in mammalian cells and plays vital roles in many cellular processes, including lysosome exocytosis (4, 5, 6), lysosomal biogenesis (7, 8), lysosomal motility (9), membrane trafficking (10, 11), and autophagy (12, 13, 14, 15, 16, 17, 18). While its role in Ca^2+^ signaling is well established, the involvement of TRPML1 in the transport of other ions, particularly Zn^2+^, has only recently gained attention. TRPML1 knockdown leads to lysosomal Zn^2+^ accumulation (19, 20), and our laboratory has shown that TRPML1 activation promotes Zn^2+^ release from late endosomes and lysosomes into the cytosol (21). Importantly, the TRPML1-mediated Zn^2+^ mobilization appears to be a distinctive phenomenon observed specifically in neurons (21), suggesting a unique role for Zn^2+^ signaling in neurophysiological processes and highlighting the importance of TRPML1-mediated Zn^2+^ release alongside its role in Ca^2+^ homeostasis.

Loss-of-function mutations in *MCOLN1*, the gene encoding TRPML1, are the genetic cause of mucolipidosis type IV (MLIV), a lysosomal storage disorder that primarily affects the central nervous system (CNS) (22). Currently, clinical care for MLIV patients is limited to symptom management, but CNS-targeted adeno-associated virus gene transfer of *MCOLN1* was recently shown to improve brain pathology and ameliorate motor decline in *MCOLN*1^−/−^ mice (23). Numerous TRPML1 mutations have been identified and categorized by clinical severity (Table 1), with prior studies showing that some of these mutations cause TRPML1 to mislocalize or exhibit altered ion permeability (1, 24, 25, 26). There has yet to be a comprehensive study that characterizes and compares the subcellular localization and ion permeabilities of the majority of TRPML1 patient mutations. Furthermore, a TRPM1 mutant that lacks Zn^2+^ permeability while maintaining Ca^2+^ transport has not yet been described. Such a mutant could serve as a useful tool to distinguish the individual contributions of Ca^2+^ and Zn^2+^ in TRPML1-mediated physiological and pathological processes.

**Table 1:** List of TRPML1 mutants derived from MLIV disease patients in order of increasing severity

Genotype	Severity	Mutation type	Region	Localization
WT	N/A	N/A	N/A	Lysosome
DDKK	N/A	N/A	Selectivity filter	Lysosome
F408Δ	Mildest	Amino acid deletion	Linker helix S4 & S5	Lysosome
L106P	Milder	Missense	I–II linker	ER
C166F	Milder	Missense	I–II linker	Punctate/ER
T232P	Milder	Missense	I–II linker	ER
D362Y	Mild	Missense	S3 helix	ER
R403C	Mild	Missense	S4 helix	ER
V446L	More severe	Missense	S5 helix	ER
L447P	More severe	Missense	S5 helix	Lysosome
F465L	More severe	Missense	Pore loop	Lysosome
C463Y	More severe	Missense	Pore loop	Lysosome

N/A, not available.

Alongside TRPML1, TRPML2 (encoded by the *MCOLN2* gene) and TRPML3 (encoded by the *MCOLN3* gene) constitute the TRPML protein family in mammals, sharing approximately 75% amino acid sequence identity (27, 28). Unlike TRPML1, TRPML2 and TRPML3 are not ubiquitously expressed. TRPML2 is highly expressed in immune cells and likely plays a crucial role in innate immunity, although it is not currently associated with any known disease phenotype (29, 30). TRPML3 expression is restricted to specific cell types, including skin melanocytes, cochlear cells, lung macrophages, olfactory sensory neurons, and cells of the kidney ducts (31, 32). Unlike TRPML1 and TRPML2, TRPML3 is inhibited by acidic luminal pH, suggesting distinct endolysosomal functions (33). It has also been shown to inhibit mechanistic target of rapamycin through a positive feedback mechanism and to participate in immune function (34, 35). Although both TRPML2 and TRPML3 have been shown to permeate Ca^2+^, their ability to permeate Zn^2+^ has not been investigated.

Endogenous TRPML1 contains a dileucine motif that directs it to lysosomes (22, 36). Although TRPML2 does not share this exact motif, it has been shown to localize to lysosomes, early endosomes, and long tubular recycling endosomes in HeLa cells and human embryonic kidney 293 (HEK293) cells (37, 38, 39). TRPML3, however, lacks any analogous motif, and some studies have reported its localization to endoplasmic reticulum (ER) membranes in HEK293 cells (40). Other studies have contradicted this, showing endosomal localization of TRPML3 in retinal pigment epithelial ARPE-19 cells, suggesting that TRPML3 also resides on endolysosomal membranes (41). Some studies reporting endosomal localization also show TRPML3 on both ER and punctate structures in CL4 cells (42) and HeLa cells (43), although ER makers were omitted from these analyses. Interestingly, coexpression of TRPML1 or TRPML2 with TRPML3 can relocate TRPML3 to lysosomes in HEK293 cells (40). Additional studies are necessary to clarify the subcellular localization of TRPML2 and TRPML3.

To minimize variability introduced by cell-type–specific trafficking mechanisms and to facilitate reliable imaging-based assessment of localization and ion permeability, we conducted all experiments in HeLa cells because of their well-characterized properties, high transfection efficiency, and common use in TRPML studies. Using live-cell confocal microscopy, we demonstrate that in HeLa cells, nearly all TRPML1 patient-derived MLIV mutants, except for one mild variant, exhibit little to no permeability to either Ca^2+^ or Zn^2+^, and this loss of ion transport is not because of altered trafficking of TRPML1. We further show that, although TRPML3 localizes to the ER, TRPML1 and TRPML3 share similar ion permeability profiles, whereas TRPML2 lacks Zn^2+^ permeability. Finally, we introduce a metal-selective TRPML1 mutant, I468V, designed based on sequence homology with TRPML2, which selectively permeates Ca^2+^ but not Zn^2+^. Taken together, our studies provide new insights into the permeation properties of TRPML1-3 and TRPML1 disease-associated mutants and establish a foundation for engineering selective ion channel variants.

## Results

### Subcellular localization of TRPML1-3

**Figure 1:**
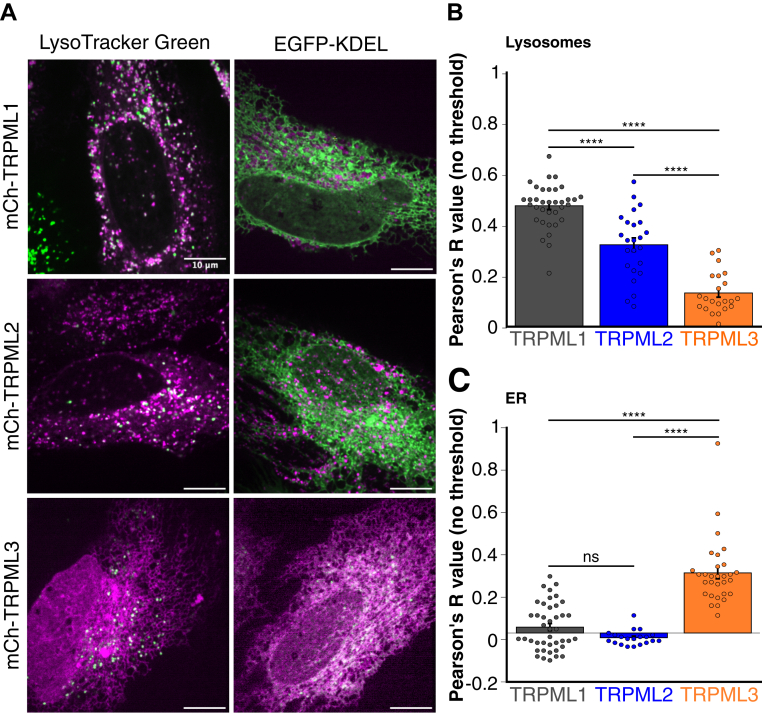
**Subcellular localization of TRPML1-3.***A*, representative images of HeLa cells that were transfected with mCherry-tagged TRPML1, TRPML2, and TRPML3. HeLa cells were either costained with LysoTracker Green (*left panels*) or cotransfected with EGFP-KDEL (*right panels*). The scale bar represents 10 μm. *B*, colocalization analysis of mCherry-TRPML1-3 to lysosomes using LysoTracker Green. Mean Pearson’s *R* value. Data are presented as mean ± SEM, and individual data points are technical replicates. N = 35, TRPML1; N = 24, TRPML2; and N = 22, TRPML3. One-way ANOVA with Tukey's honest significant difference multiple comparison test. ∗∗∗∗*p* < 0.0001. *C*, colocalization analysis of mCherry-TRPML1-3 to the endoplasmic reticulum using EGFP-KDEL. Mean Pearson’s *R* value. Data are presented as mean ± SEM, and individual data points are technical replicates. N = 42, TRPML1; N = 20, TRPML2; and N = 30, TRPML3. One-way ANOVA with Tukey's honest significant difference multiple comparison test. ∗∗∗∗*p* < 0.0001, ns not significant. EGFP, enhanced GFP; TRPML, transient receptor potential mucolipin.

Reports of TRPML2 and TRPML3 localization have been inconsistent across cell types, with TRPML3 variably observed on endolysosomal and ER membranes and TRPML2 localizing to both early endosomes and lysosomes. Here, we directly compared the subcellular localization of TRPML1-3 in the same cell type under identical experimental conditions. mCherry-tagged TRPML1, TRPML2, and TRPML3 were costained with LysoTracker Green to assess lysosomal colocalization or cotransfected with enhanced GFP (EGFP)-KDEL, a green ER marker (Fig. 1*A*). All three TRPMLs colocalized with lysosomes, although TRPML3 did so to a significantly lesser extent (Fig. 1, *A* and *B*). Notably, TRPML3 was the only channel to show ER colocalization, doing so nearly twice as strongly as its colocalization to lysosomes (Fig. 1, *A* and *C*). These results suggest that in HeLa cells, TRPML1 and TRPML2 primarily localize to lysosomes, whereas TRPML3 localizes to both lysosomes and the ER, with a preference for the ER.

### MLIV-associated TRPML1 patient mutants show variable subcellular localization

**Figure 2:**
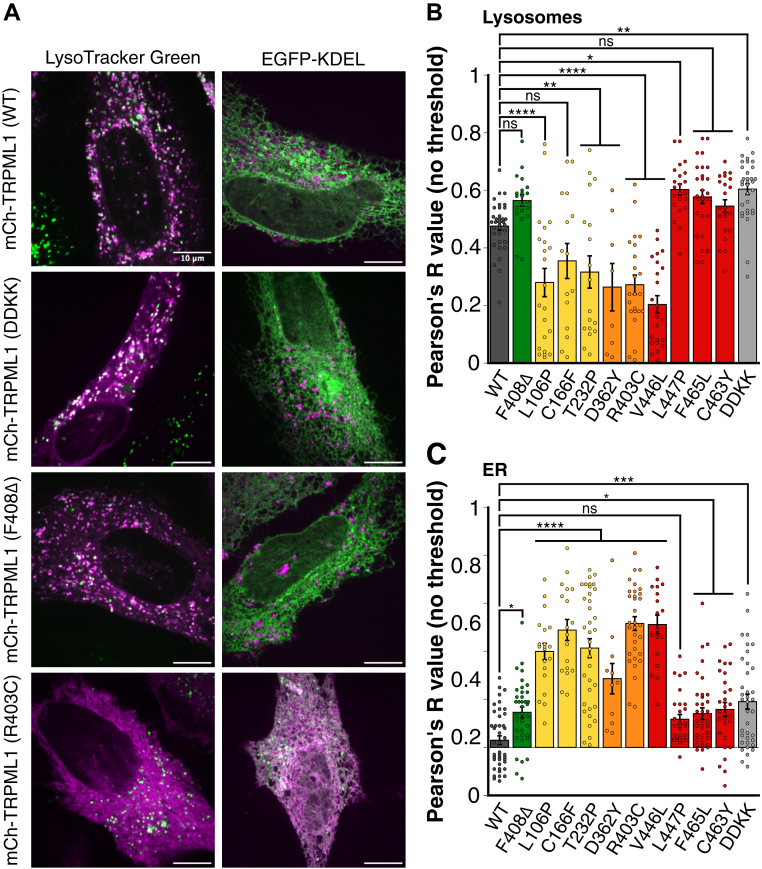
**TRPML1 patient mutants show variable subcellular localization.***A*, representative images of HeLa cells that were transfected with mCherry-tagged TRPML1 wildtype and mutants DDKK, F408Δ, and R403C. HeLa cells were either costained with LysoTracker Green (*left panels*) or cotransfected with EGFP-KDEL (*right panels*). The scale bar represents 10 μm. The representative mCherry-TRPML1 wildtype image is the same source image shown in Figure 1*A*, reused intentionally to illustrate wildtype localization under comparable imaging conditions. *B*, colocalization analysis of mCherry-TRPML1 wildtype and mutants to lysosomes using LysoTracker Green. Mean Pearson’s *R* value. Mutants are color coded and listed in the order of increasing severity. Data are presented as mean ± SEM, and individual data points are technical replicates. N = 35, wildtype; N = 21, F408Δ; N = 22, L106P; N = 15, C166F; N = 18, T232P; N = 8, D362Y; N = 23, R403C; N = 23, V446L; N = 22, L447P; N = 28, F465L; N = 22, C463Y; and N = 32, DDKK. One-way ANOVA with Dunnett’s multiple comparison to wildtype TRPML1. ∗∗∗∗*p* < 0.0001, ∗∗*p* < 0.01, ∗*p* < 0.05, and ns, not significant. *C*, colocalization analysis of mCherry-TRPML1 wildtype and mutants to the ER using EGFP-KDEL. Mean Pearson’s *R* value. Data are presented as mean ± SEM, and individual data points are technical replicates. N = 42, wildtype; N = 37, F408Δ; N = 20, L106P; N = 18, C166F; N = 36, T232P; N = 11, D362Y; N = 32, R403C; N = 18, V446L; N = 29, L447P; N = 36, F465L; N = 29, C463Y; and N = 37, DDKK. One-way ANOVA with Dunnett’s multiple comparison to wildtype TRPML1. ∗∗∗∗*p* < 0.0001, ∗∗∗*p* < 0.001, ∗*p* < 0.05, and ns not significant. EGFP, enhanced GFP; TRPML, transient receptor potential mucolipin.

Prior studies have reported that several TRPML1 mutants mislocalize to different organelles within the cell, such as the ER. We therefore wanted to determine the localization of the 10 TRPML1 mutants derived from MLIV patients (Table 1). To assess lysosomal colocalization, mCherry-TRPML1 mutants were costained with LysoTracker Green (Fig. 2*A*). Mutants F408Δ, L447P, F465L, and C463Y colocalized with lysosomes to a similar extent as wildtype TRPML1 (Fig. 2, *A* and *B*). Since multiple mutants appear to localize with the ER, we cotransfected the mCherry-TRPML1 constructs with EGFP-KDEL (Fig. 2*A*). Mutants L106P, C166F, T232P, D362Y, R403C, and V446L all showed high ER colocalization and a significant decrease in lysosomal colocalization (Fig. 2, *A* and *C*). Although mutants F408Δ, F465L, and C463Y were not statistically different from wildtype TRPML1 in lysosomal colocalization, they were statistically different in ER colocalization. These results suggest that TRPML1 mutations in MLIV patients can alter not only ion permeability but also subcellular localization. Both the mildest mutant, F408Δ, and the most severe mutants, L447P, F465L, and C463Y, localize to lysosomes. In contrast, the ER localized mutants are associated with a wide range of disease severity. These findings suggest that subcellular localization alone does not determine the disease severity in MLIV.

### Comparing Ca^2+^ and Zn^2+^ permeability among TRPML1, TRPML2, and TRPML3

**Figure 3:**
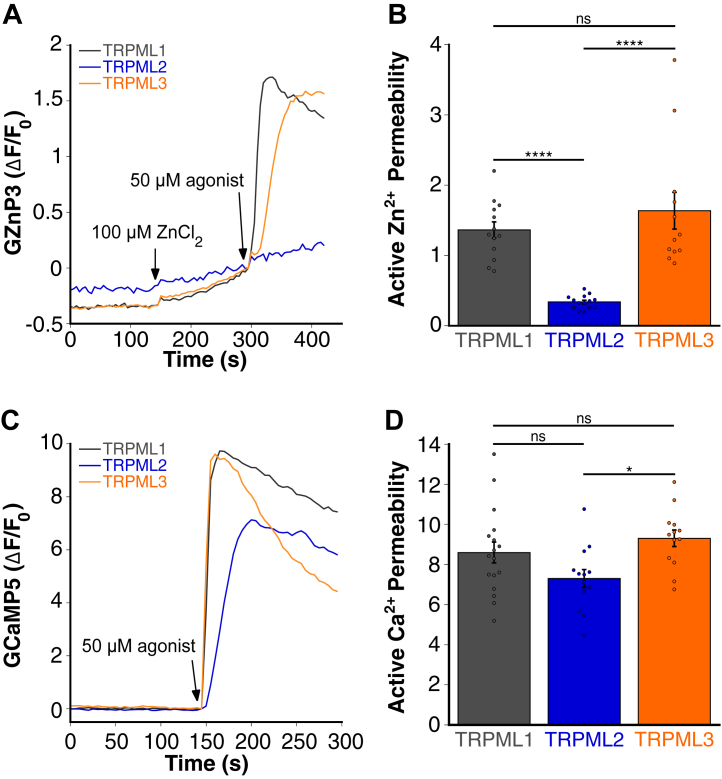
**TRPML2 is impermeable to Zn^2+^, whereas TRPML3 shares similar permeability with TRPML1.***A*, representative traces of agonist-mediated Zn^2+^ uptake (GZnP3 ΔF/F_0_) over time in HeLa cells overexpressing mCherry-tagged TRPML1, TRPML2, and TRPML3 on the plasma membrane (PM) cotransfected with GZnP3 in a calcium-free buffer. Cells overexpressing TRPML1 and TRPML3 were incubated with 100 μM ZnCl_2_ and treated with 50 μM ML-SA1 to induce TRPML1/3 channel opening, whereas cells overexpressing TRPML2 were treated with 50 μM ML2-SA1. *B*, quantification of agonist-mediated Zn^2+^ uptake through TRPML1-3 channels based on the peak ΔF/F_0_ values of GZnP3 following ML-SA1 or ML2-SA1 activation. Traces were normalized to the timepoint before agonist addition. Data are presented as mean ± SEM, and individual data points are technical replicates. N = 13, TRPML1; N = 14, TRPML2; and N = 12, TRPML3. One-way ANOVA with Tukey's honest significant difference multiple comparison. ∗∗∗∗*p* < 0.0001; ns, not significant. *C*, representative traces of agonist-mediated Ca^2+^ uptake (GCaMP5 ΔF/F_0_) over time in HeLa cells overexpressing mCherry-tagged TRPML1, TRPML2, and TRPML3 on the PM cotransfected with GCaMP5 in calcium-containing buffer. Cells were treated with 50 μM ML-SA1 or 50 μM ML2-SA1 to induce TRPML1-3 channel opening. *D*, quantification of agonist-mediated Ca^2+^ uptake through TRPML1-3 channels based on the peak ΔF/F_0_ values of GCaMP5 following ML-SA1 or ML2-SA1 activation. Traces were normalized to the timepoint before agonist addition. Data are presented as mean ± SEM, and individual data points are technical replicates. N = 17, TRPML1; N = 13, TRPML2; and N = 13, TRPML3. One-way ANOVA with Tukey's honest significant difference multiple comparison test. ∗*p* < 0.05; ns, not significant. TRPML, transient receptor potential mucolipin.

Previous studies of TRPML1 permeability have been conducted using electrophysiological recordings (1, 3, 10, 44, 45, 46, 47, 48, 49, 50, 51). However, these approaches are technically challenging, often have low throughput, and are difficult to perform in live cells. Here, we employed genetically encoded sensors—GZnP3 for Zn^2+^ and GCaMP5 for Ca^2+^—to directly monitor cytosolic cation dynamics in live cells. We investigated the agonist-mediated rates of Zn^2+^ and Ca^2+^ permeability of all three members of the mucolipin family, TRPML1, TRPML2, and TRPML3. We used different agonists to ensure maximum activation. TRPML1 and TRPML3 were stimulated with ML-SA1, which is known to activate all TRPML channels. TRPML2 was activated with ML2-SA1, a more selective synthetic agonist of TRPML2 compared with ML-SA1 (30, 52). We monitored changes in fluorescence of GZnP3 and GCaMP5 in HeLa cells cotransfected with mCherry-tagged TRPML constructs. Overexpression of TRPML channels causes their partial localization to the plasma membrane (53, 54), allowing us to measure their ion permeability *via* monitoring cation influx. To maximize the sensitivity of this *in situ* assay, we utilized 100 μM ZnCl_2_ because limited Zn^2+^ influx can be detected at 5 μM ZnCl_2_ (Fig. S1). Upon ML-SA1 activation, TRPML3 showed robust Zn^2+^ and Ca^2+^ influx comparable to that of TRPML1 (Fig. 3, *A*–*D*). Zn^2+^ and Ca^2+^ permeability were quantified as changes in GZnP3 or GCaMP5 fluorescence (ΔF/F_0_), respectively. In contrast, ML2-SA1 activation of TRPML2 resulted in no active Zn^2+^ influx compared with TRPML1 and TRPML3 (Fig. 3, *A* and *B*), indicating that TRPML2 is not permeable to Zn^2+^. TRPML2 Ca^2+^ permeability was similar, though slightly lower, than TRPML1 and TRPML3 (Fig. 3, *C* and *D*). These data suggest that TRPML1 and TRPML3 are permeable to both Zn^2+^ and Ca^2+^, whereas TRPML2 selectively permeates Ca^2+^.

### TRPML1 patient mutants exhibit reduced permeability to Zn^2+^ and Ca^2+^

**Figure 4:**
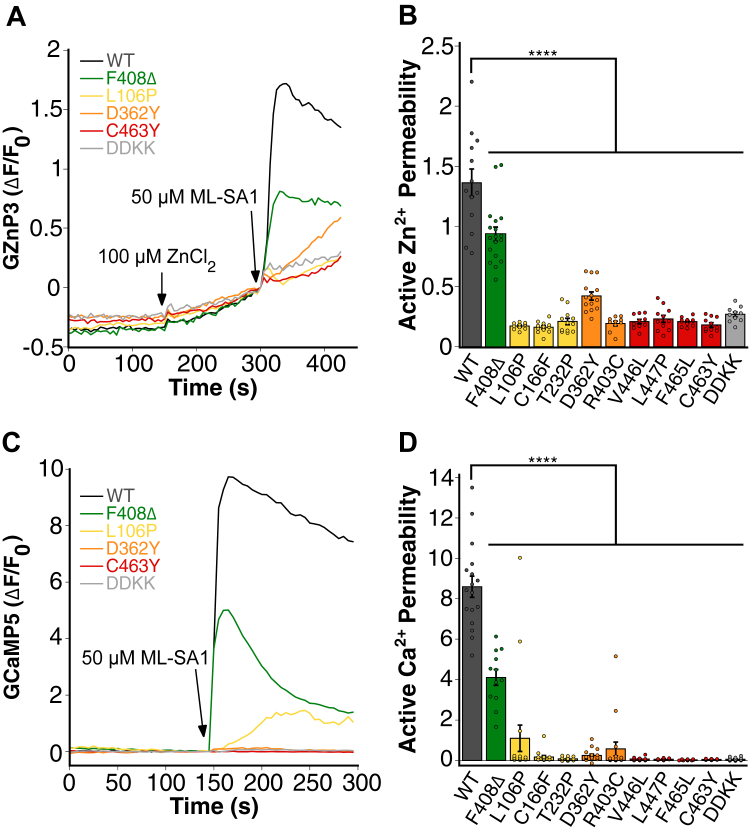
**TRPML1 patient mutants exhibit reduced permeability to Zn^2+^ and Ca^2+^.***A*, representative traces of agonist-mediated Zn^2+^ uptake (GZnP3 ΔF/F_0_) over time in HeLa cells overexpressing mCherry-tagged wildtype, dominant-negative, and select TRPML1 mutants on the plasma membrane (PM) cotransfected with GZnP3 in calcium-free buffer. Cells were incubated with 100 μM ZnCl_2_ and treated with 50 μM ML-SA1 to induce TRPML1 channel opening. *B*, quantification of agonist-mediated Zn^2+^ uptake through TRPML1 channels based on the peak ΔF/F_0_ values of GZnP3 following ML-SA1 activation. Traces were normalized to the timepoint before ML-SA1 addition. Mutants are color coded and listed in the order of increasing severity. Data are presented as mean ± SEM, and individual data points are technical replicates. N = 13, wildtype; N = 18, F408Δ; N = 10, L106P; N = 12, C166F; N = 12, T232P; N = 14, D362Y; N = 10, R403C; N = 10, V446L; N = 11, L447P; N = 12, F465L; N = 11, C463Y; and N = 10, DDKK. ∗∗∗∗*p* < 0.0001; ns, not significant. *C*, representative traces of agonist-mediated Ca^2+^ uptake (GCaMP5 ΔF/F_0_) over time in HeLa cells overexpressing mCherry-tagged wildtype, dominant-negative, and select TRPML1 mutants on the PM cotransfected with GCaMP5 in calcium-containing buffer. Cells were treated with 50 μM ML-SA1 to induce TRPML1 channel opening. *D*, quantification of agonist-mediated Ca^2+^ uptake through TRPML1 channels based on the peak ΔF/F_0_ values of GCaMP5 following ML-SA1 activation. Traces were normalized to the timepoint before ML-SA1 addition. Data are presented as mean ± SEM, and individual data points are technical replicates. N = 17, wildtype; N = 12, F408Δ; N = 17, L106P; N = 15, C166F; N = 17, T232P; N = 17, D362Y; N = 16, R403C; N = 12, V446L; N = 10, L447P; N = 12, F465L; N = 11, C463Y; and N = 15, DDKK. One-way ANOVA with Dunnett’s multiple comparison to wildtype TRPML1. ∗∗∗∗*p* < 0.0001; ns, not significant. TRPML, transient receptor potential mucolipin.

Next, we investigated Zn^2+^ and Ca^2+^ permeability of TRPML1 mutants derived from MLIV patients using the same experimental approach described above. In the presence of 100 μM ZnCl_2_, GZnP3 fluorescence increased gradually. Upon activation with ML-SA1, cells expressing wildtype TRPML1 exhibited a rapid and robust enhancement in GZnP3 signal (Fig. 4*A*), indicating efficient Zn^2+^ influx *via* TRPML1. In contrast, all TRPML1 patient-derived mutants displayed a significant reduction in agonist-mediated Zn^2+^ permeability. TRPML1 DDKK, a dominant-negative mutant of TRPML1 that incorporates two lysine residues in place of aspartate residues in the channel’s pore to block its uptake of cations (55), exhibited no response. The mildest mutant, F408Δ, decreased TRPML1’s permeability to Zn^2+^ by 31%. The remaining mutants showed little to no response to ML-SA1, with mutants V446L, L447P, F465L, C463Y, and T232P replicating the effects of TRPML1 DDKK. These data demonstrate that all disease-associated mutations in TRPML1 severely impair agonist-mediated Zn^2+^ permeability. When measuring Ca^2+^ dynamics, GCaMP5 fluorescence was significantly increased upon activation with ML-SA1 in cells expressing wildtype TRPML1 (Fig. 4*C*). Among the patient mutants, F408Δ maintained 48% of the wildtype Ca^2+^ permeability, whereas all other mutants resulted in an almost complete loss of Ca^2+^ permeability. These results suggest that TRPML1 patient mutations significantly compromise the channel’s ability to conduct both Zn^2+^ and Ca^2+^.

### Some TRPML1 patient mutants generate constitutive permeability

**Figure 5:**
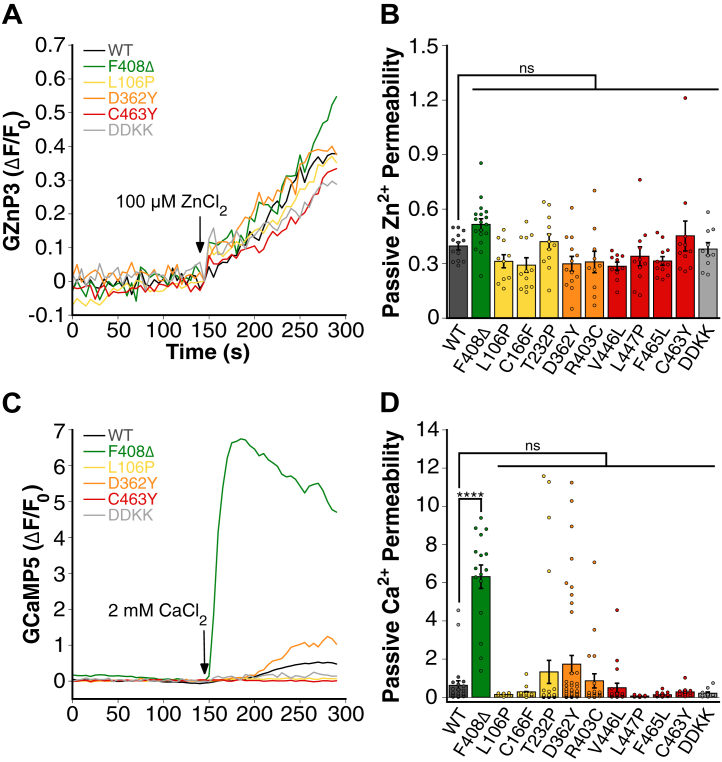
**Some TRPML1 patient mutants are constitutively permeable to Ca^2+^ but not to Zn^2+^.***A*, representative traces of passive Zn^2+^ uptake (GZnP3 ΔF/F_0_) over time in HeLa cells overexpressing mCherry-tagged wildtype, dominant-negative, and select TRPML1 mutants on the plasma membrane (PM) cotransfected with GZnP3 in calcium-free buffer. Cells were incubated with 100 μM ZnCl_2_, without ML-SA1. *B*, quantification of passive Zn^2+^ uptake through TRPML1 channels based on the fluorescence amplitudes of GZnP3 following Zn^2+^ addition. Data are presented as mean ± SEM, and individual data points are technical replicates. N = 13, wildtype; N = 18, F408Δ; N = 10, L106P; N = 12, C166F; N = 12, T232P; N = 13, D362Y; N = 10, R403C; N = 10, V446L; N = 11, L447P; N = 12, F465L; N = 11, C463Y; and N = 10, DDKK. One-way ANOVA with Dunnett’s multiple comparison to wildtype TRPML1. ns, not significant. Traces were normalized to the timepoint before ZnCl_2_ addition. Mutants are color coded and listed in the order of increasing severity. *C*, representative traces of passive Ca^2+^ uptake (GCaMP5 ΔF/F_0_) over time in HeLa cells overexpressing mCherry-tagged wildtype, dominant-negative, and select TRPML1 mutants on the PM cotransfected with GCaMP5 in calcium-free buffer. Cells were incubated with 2 mM CaCl_2_, without ML-SA1. *D*, quantification of passive Ca^2+^ uptake through TRPML1 channels based on the fluorescence amplitudes of GCaMP5 following Ca^2+^ addition. Traces were normalized to the timepoint before CaCl_2_ addition. Data are presented as mean ± SEM, and individual data points are technical replicates. N = 24, wildtype; N = 16, F408Δ; N = 9, L106P; N = 10, C166F; N = 31, T232P; N = 43, D362Y; N = 21, R403C; N = 20, V446L; N = 11, L447P; N = 16, F465L; N = 11, C463Y; and N = 10, DDKK. One-way ANOVA with Dunnett’s multiple comparison to wildtype TRPML1. ∗∗∗∗*p* < 0.0001; ns, not significant. TRPML, transient receptor potential mucolipin.

To further study the effects of TRPML1 mutations, we next compared their passive, or agonist-independent, ion permeability. Specifically, we sought to determine whether TRPML1 variants could mediate cation influx under baseline conditions in the absence of agonist activation. Across all tested TRPML1 variants, there were no significant differences in GZnP3 fluorescence after the addition of 100 μM ZnCl_2_ compared with the wildtype (Fig. 5, *A–B*). In contrast, F408Δ exhibited a significant increase in Ca^2+^ uptake, indicating that this mutant is passively permeable to Ca^2+^. All other mutants showed no significant passive Ca^2+^ uptake compared with wildtype (Fig. 5, *C* and *D*). Compared with Zn^2+^, the passive Ca^2+^ signals displayed large variations, which are likely caused by Ca^2+^ oscillations in cells with changes of extracellular Ca^2+^. These data suggest that the F408Δ mutant is a leaky channel selectively permeable to Ca^2+^.

### Generating a TRPML1 mutant that is selectively permeable to Ca^2+^ and not to Zn^2+^

**Figure 6:**
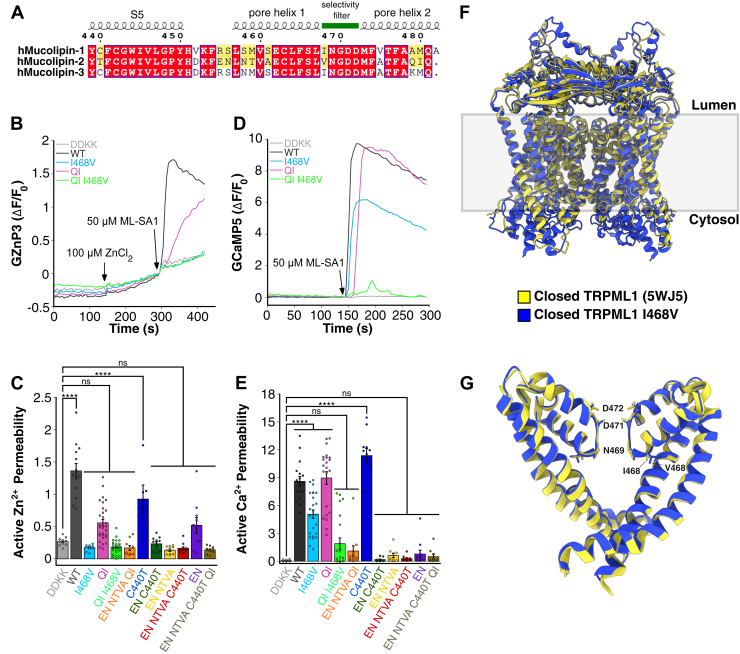
**Generating a TRPML1 mutant that is selectively permeable to Ca^2+^ and not to Zn^2+^.***A*, multiple sequence alignment of TRPML1-3 highlighting sequence differences in the S5, PH1, selectivity filter, and PH2 regions. hMucolipin1 (human, UniProt ID: Q9GZU1), hMucolipin2 (human, UniProt ID: Q8IZK6), hMucolipin3 (human, UniProt ID: Q8TDD5). *B*, representative traces of agonist-mediated Zn^2+^ uptake (GZnP3 ΔF/F_0_) over time in HeLa cells overexpressing mCherry-tagged wildtype, dominant-negative, and select TRPML1 mutants on the plasma membrane (PM) cotransfected with GZnP3 in calcium-free buffer. Cells were incubated with 100 μM ZnCl_2_ and treated with 50 μM ML-SA1 to induce TRPML1 channel opening. *C*, quantification of agonist-mediated Zn^2+^ uptake through TRPML1 channels based on the peak ΔF/F_0_ values of GZnP3 following ML-SA1 activation. Traces were normalized to the timepoint before ML-SA1 addition. Data are presented as mean ± SEM, and individual data points are technical replicates. N = 10, DDKK; N = 13, wildtype; N = 10, I468V; N = 30, QI; N = 32, QI I468V; N = 10, EN NTVA QI; N = 13, C440T; N = 11, EN C440T; N = 10, EN NTVA; N = 11, EN NTVA C440T; N = 10, EN; and N = 11, EN NTVA C440T QI. One-way ANOVA with Dunnett’s multiple comparison to TRPML1 DDKK. ∗∗∗∗*p* < 0.0001; ns, not significant. *D*, representative traces of passive Ca^2+^ uptake (GCaMP5 ΔF/F_0_) over time in HeLa cells overexpressing mCherry-tagged wildtype, dominant-negative, and select TRPML1 mutants on the PM cotransfected with GCaMP5 in calcium-containing buffer. Cells were treated with 50 μM ML-SA1 to induce TRPML1 channel opening. *E*, quantification of agonist-mediated Ca^2+^ uptake through TRPML1 channels based on the peak ΔF/F_0_ values of GCaMP5 following ML-SA1 activation. Traces were normalized to the timepoint before ML-SA1 addition. Data are presented as mean ± SEM, and individual data points are technical replicates. N = 15, DDKK; N = 17, wildtype; N = 25, I468V; N = 23, QI; N = 21, QI I468V; N = 12, EN NTVA QI; N = 11, C440T; N = 10, EN C440T; N = 9, EN NTVA; N = 12, EN NTVA C440T; N = 11, EN; and N = 10, EN NTVA C440T QI. One-way ANOVA with Dunnett’s multiple comparison test to TRPML1 DDKK. ∗∗∗∗*p* < 0.0001; ns, not significant. *F*, structural comparison between closed TRPML1 (Protein Data Bank code: 5WJ5) in *yellow* and closed TRPML1 I468V predicted by AlphaFold 3 in *blue*. Residue 468 in both structures is shown as *sticks*. *G*, the closed ion-conduction pore of TRPML1 and TRPML1 I468V, with only two diagonal subunits shown for clarity. The following pore-lining residues are shown as *sticks*: I468 (*yellow*), V468 (*blue*), N469, D471, and D472. PH, pore helix; TRPML, transient receptor potential mucolipin.

Given that TRPML2 retains robust Ca^2+^ conductance while lacking permeability to Zn^2+^, we aimed to generate a TRPML1 mutant that mimics this selective ion permeability. Specifically, we wanted to engineer a TRPML1 variant selectively permeable to Ca^2+^ but not to Zn^2+^. To achieve this, we performed a sequence alignment of TRPML1, TRPML2, and TRPML3. The amino acid sequences of TRPML1-3 were aligned, and regions where TRPML1/3 and TRPML2 differed were identified, focusing on regions critical for ion selectivity and permeation (Fig. 6*A*). The TRPML1 luminal pore is formed by the fifth transmembrane helix (S5), S6, pore helix (PH1), selectivity filter, and pore helix 2 (PH2) (Fig. 6*G*). Within this region, we introduced 10 mutations to mCherry-TRPML1 by substitution with TRPML2-like amino acids (Table 2). Agonist-mediated permeability assays were performed as previously described. Of the 10 mutants tested, TRPML1 QI, C440T, and EN showed higher Zn^2+^ permeability compared with nonfunctional TRPML1 DDKK, whereas the others were impermeable to Zn^2+^ (Fig. 6, *B* and *C*). In addition, TRPML1 I468V, QI, and C440T exhibited decent Ca^2+^ permeability when compared with TRPML1 DDKK (Fig. 6, *D* and *E*). Notably, the I468V mutation produced an ion selectivity that completely abolished Zn^2+^ permeability while retaining robust Ca^2+^ transport (Fig. 6, *C* and *E*). The structure of TRPML1 I468V in the closed state was predicted by AlphaFold 3 (Fig. S2) (56). When compared with wildtype TRPML1, no significant structural changes in the pore region near the selectivity filter were observed. However, the proximity of I468 to important residues, such as N469, G470, and D471, within the constricted pore regions suggests that this mutation could subtly alter the pore size when the TRPML1 channel is open (Fig. 6, *F* and *G*). Given that I468 is positioned at the corner where PH1 makes a sharp turn toward the selectivity filter, we hypothesize that substituting isoleucine with valine slightly alters the selectivity filter opening. This subtle change may restrict the passage of hydrated Zn^2+^, whose hydrated radius is slightly larger than that of hydrated Ca^2+^ (57). Further structural and computational studies will be needed to confirm this hypothesis. These findings demonstrate that we successfully engineered a TRPML1 mutant that is permeable to Ca^2+^ but not to Zn^2+^. This TRPML1 I468 mutant represents a valuable tool for dissecting the distinct physiological roles of Zn^2+^ and Ca^2+^ flux through TRPML1 channels.

**Table 2:** List of TRPML1 mutants designed based on TRPML2 sequence homology

TRPML1 mutants	Genotype	Zn^2+^ permeability	Ca^2+^ permeability
TRPML1 I468V	I468V	No	Yes
TRPML1 QI	A479Q M480I	Yes	Yes
TRPML1 QI I468V	I468V A479Q M480I	No	No
TRPML1 EN NTVA QI	R455E S456N S458N M459T S461A A479Q M480I	No	No
TRPML1 C440T	C440T	Yes	Yes
TRPML1 EN C440T	C440T R455E S456N	No	No
TRPML1 EN NTVA	R455E S456N S458N M459T S461A	No	No
TRPML1 EN NTVA C440T	C440T R455E S456N S458N M459T S461A	No	No
TRPML1 EN	R455E S456N	Yes	No
TRPML1 EN NTVA C440T QI	C440T R455E S456N S458N M459T S461A A479Q M480I	No	No

## Discussion

TRPML channels play critical roles in cellular ion homeostasis, particularly in the endolysosomal system. Disruptions in their function are implicated in diseases. Loss-of-function mutations in TRPML1 cause MLIV, an autosomal recessive lysosomal storage disorder that primarily affects the CNS and is characterized by impaired psychomotor development, developmental delay, and retinal dystrophy (22, 58, 59, 60, 61). In contrast, mutations in TRPML2 have not been directly linked to any known human diseases. However, its expression pattern suggests a role in immunity. Unlike the ubiquitous expression of TRPML1 across various tissues, TRPML2 expression is predominantly found in lymphoid and myeloid tissues, contributing to the immune function (37). While TRPML3 mutations have not been linked to human disease, a gain-of-function mutation (A419P) in mouse TRPML3 has been shown to cause significant defects, including hearing loss, vestibular dysfunction, and impaired coat pigmentation (45, 62). Understanding how these channels differ in localization, ion selectivity, and mutation sensitivity is critical for deciphering their functional mechanisms and identifying therapeutic strategies, particularly for MLIV.

Our work provides important structural and functional insights into the TRPML1 channel. Over 20 TRPML1 mutations have been identified to date, including splice, nonsense, missense, and in-frame deletion variants (10, 22, 58, 60, 63, 64, 65, 66, 67, 68). Prior work has shown that some of these mutations impair TRPML1 trafficking and localization, whereas others retain lysosomal localization but have lost ion permeability (1, 24, 25, 26). In our study, of the 10 MLIV mutants we examined, six of these, including L106P, C166F, T232P, D362Y, R403C, and V446L, failed to localize to lysosomes and instead colocalized strongly with the ER marker (Fig. 2), suggesting defects in protein folding or trafficking. The location of the mutations in these ER-retained mutants clusters in the N-terminal half of the protein, up to the S4 helix. In contrast, mutations that correctly localize to lysosomes—F408Δ, L447P, F465L, and C463Y—are situated between the S4 helix and the pore loop near the C terminus. Thus, ER-localized mutations likely impair protein trafficking because of misfolding of TRPML1, whereas pore-proximal mutations compromise channel function through altered gating or ion selectivity. All 10 mutants showed severely reduced agonist-mediated Zn^2+^ and Ca^2+^ permeability (Fig. 4). Apart from F408Δ, all mutations displayed permeability profiles similar to a nonfunctional TRPML1 DDKK mutant, suggesting near abolishment of conductance. Interestingly, we found that the F408Δ mutant is a constitutively active Ca^2+^ channel (Fig. 5). F408 lies in the S4–S5 linker, a region important for gating control. The constitutive activity of F408Δ suggests the critical role of F408 in maintaining proper gating control of the TRPML1 channel and preventing spontaneous channel opening.

All three TRPML channels are reported as nonselective cation channels, releasing cations from the endolysosomal lumen into the cytosol. Electrophysiological studies have shown that TRPML1 is permeable to Ca^2+^, Fe^2+^, and Zn^2+^ (69). Using live-cell microscopy combined with a sensitive fluorescent Zn^2+^ sensor, TRPML1-mediated Zn^2+^ release has been detected in primary hippocampal and cortical neurons (21). In addition, TRPML1-mediated Zn^2+^ release has been implicated in necrotic cell death in metastatic melanoma cells (70). Using both radiolabeled iron uptake assays and patch clamping, TRPML2 has been found to exhibit high permeability to Fe^2+^, even greater than TRPML1, whereas TRPML3 is impermeable to Fe^2+^ (1). However, a systematic comparison of their Ca^2+^ and Zn^2+^ permeability is lacking. Here, we showed that all three TRPML channels demonstrate comparable permeability to Ca^2+^, but only TRPML2 lacks permeability to Zn^2+^ (Fig. 3). These differences in metal ion selectivity might account for the distinct physiological roles of each TRPML isoform. TRPML2 is expressed in a limited range of tissues, primarily within lymphoid and myeloid cells (37). Notably, TRPML2 has been reported to be uniquely sensitive to hypotonic challenge and mechanical stimuli, suggesting an important role for TPRML2-mediated lysosomal Ca^2+^ release in fast-recycling processes of activated innate immune cells (71). Its more selective ion permeability profile likely corresponds to this distinctive tissue expression. In contrast, TRPML1’s ubiquitous expression and ability to transport several ions, including Zn^2+^, may explain its involvement in diverse biological processes and its association with severe diseases such as MLIV. Together, these findings suggest that differences in metal ion handling between TPRML isoforms reflect specialized physiological functions across cell types and tissue.

To further investigate the structural basis for Zn^2+^ selectivity, we took advantage of the ease of fluorescent imaging, high sensitivity of genetically encoded sensors, and plasma membrane localization of overexpressed TRPML1 channels (53, 54). We screened a panel of 10 TRPML1 mutants for their Ca^2+^ and Zn^2+^ permeability. One amino acid difference at position 468, a valine in TRPML2 and an isoleucine in TRPML1 and TRPML3, stood out. Located in PH1 adjacent to the selectivity filter, this residue contributes to a hydrophobic cavity involved in ML-SA1 binding (72). We found that TRPML1 I468V selectively abolished Zn^2+^ sensitivity while preserving Ca^2+^ transport (Fig. 6). The selectivity filter of TRPML1 has the sequence 469NGDDM and contains multiple ion binding sites (Fig. 6, *A* and *G*). D471 and D472 are acidic residues at the luminal side that stabilize the filter and create a negative electrostatic trap for divalent ion passage (73). The carbonyl oxygen atoms of N469 are 1.8 Å further apart in the ML-SA1-bound open state compared with the state (72). Also, 2.4 Å expansion is predicted at the narrowest constriction of G470 when TRPML1 is bound with its endogenous agonist PI(3,5)P2 (74). Furthermore, agonist binding eases the restriction of the lower gate and induces a structural rearrangement of PH1, forming a new hydrophilic bond between Y507 in S6 and N469 in PH1, slightly opening the selectivity filter (72). Given the proximity of I468 to this region, we hypothesize that substituting isoleucine with valine hinders the normal opening of the selectivity filter, perhaps by reducing side-chain bulk. Altered electrostatic interactions or pore size may block the passage of hydrated Zn^2+^ ions, who has a larger radius than hydrated Ca^2+^ ions (57). These subtle electrostatic and steric alterations therefore provide a plausible explanation for how Zn^2+^ transport can be selectively impaired without affecting Ca^2+^ conductance.

Our data illustrated a new molecular basis of MLIV diseases. First, the F408Δ mutant, which is associated with the mildest clinical symptoms, retains partial permeability to both Ca^2+^and Zn^2+^. This suggests that residual ion permeability may be sufficient to support partial lysosomal function and mitigate disease severity. Importantly, this mutant also demonstrates constitutive Ca^2+^ conductance, but not Zn^2+^ conductance, suggesting that a dysregulated gain of function is not detrimental to brain function. It is possible that a certain degree of Ca^2+^ leakage from TRPML1 may be tolerable. Second, most of the patient-derived TRPML1 mutants completely lose their permeability to Ca^2+^ and Zn^2+^, consistent with a loss-of-function mechanism. However, they vary significantly in subcellular localization, with some retained in the ER and others properly targeted to lysosomes. Interestingly, we observed a correlation between lysosomal localization and more severe clinical phenotypes. Mutants, such as L447P, C463Y, and F465L, which localize to lysosomes but are nonfunctional, are associated with the most severe symptoms. In contrast, mutants retained in the ER, such as L106F, C166F, and T232P, tend to correlate with milder symptoms. This finding is consistent with a previous report in which a patient with a homozygous null mutation, which results in the complete absence of TRPML1 protein, exhibited relatively mild neurological symptoms (75). All these results suggest that nonfunctional TRPML1 proteins that reach the lysosome may be more detrimental than the complete loss of the protein in lysosomes. One possible explanation is that lysosomal-localized, nonfunctional TRPML1 mutants may exert a dominant-negative effect, interfering with the function of other TRPML isoforms through subunit interactions. These findings highlight the need for future studies to clarify how distinct TRPML1 mutations affect neuronal function and contribute to the spectrum of clinical outcomes in MLIV.

## Experimental procedures

### DNA constructs

Plasmids were created by molecular cloning and verified by sequencing. mCherry was fused to TRPML1, TRPML2, and TRPML3. Site-directed mutagenesis was used to introduce the following mutations to mCherry-TRPML1 to create the MLIV patient mutants: F408Δ, L106P, C166F, T232P, D362Y, R403C, V446L, L447P, F465L, and C463Y. Site-directed mutagenesis was used to introduce the following mutations to mCherry-TRPML1 to create the engineered mutants: C440T, R455E, S456N, S458N, M459T, S461A, I468V, A479Q, and M480I. All constructs were in the pcDNA3.1+ vector.

### Cell culture and transfection

HeLa cells were maintained in Dulbecco’s modified Eagle’s medium with 10% fetal bovine serum at 37 °C, 5% CO_2_. Cells were transfected at ∼40% to 50% confluency. For each transfection reaction, 3 μl polyethyleneimine transfection reagent and 1 to 1.25 μg DNA were mixed in 250 μl Opti-MEM. Reactions were incubated at room temperature for 25 min before direct addition to one imaging dish containing the cells. Imaging dishes were maintained at 37 °C until imaging.

### Mammalian cell imaging

Cells were imaged 24 to 48 post-transfection, and cells were washed three times with the indicated buffer immediately before imaging. All imagings were performed on an inverted Nikon/Solamere CSUX1 spinning disc confocal microscope with a 40× 1.4 numerical aperture (NA) oil immersion objective and 1.0× OptiVar for permeability studies or a 60× 1.4 NA oil immersion objective and 1.5× OptiVar for colocalization. Data were collected using MicroManager software. All experiments were done at room temperature. Acquisition times were 5 s for Ca^2+^ and Zn^2+^ imaging with exposure times of 200 ms.

### Recording Ca^2+^ and Zn^2+^ permeability of TRPML1-3

HeLa cells overexpressing mCherry-tagged TRPML1, TRPML2, or TRPML3 were transfected with GZnP3 or GCaMP5 for Zn^2+^ and Ca^2+^ experiments, respectively. To test Zn^2+^ permeability, cells were imaged in calcium-free phosphate-free HEPES-buffered Hank's Balanced Salt Solution (HHBSS) buffer (pH 7.4), which contains 5.4 mM KCl, 1.1 mM MgCl_2_.6H_2_O, 137 mM NaCl, 16.8 mM d-glucose, and 30 mM Hepes. After collecting a baseline signal for 2.5 min, cells were treated with 100 μM ZnCl_2_ to allow for passive Zn^2+^ permeation through the channel. After 2.5 min, 50 μM ML-SA1 (or ML2-SA1 for TRPML2) was added to activate the channel. Passive and active Ca^2+^ permeability were measured in separate experiments. For passive Ca^2+^ permeability, the cells were imaged in calcium-free phosphate-free HHBSS buffer (pH 7.4). The baseline signal was collected for 2.5 min before treating cells with 2 mM CaCl_2_ for 2.5 min. For active Ca^2+^ permeability, cells were imaged in phosphate-free HHBSS buffer (pH 7.4), which contains 1.26 mM CaCl_2,_ 5.4 mM KCl, 1.1 mM MgCl_2_.6H_2_O, 137 mM NaCl, 16.8 mM d-glucose, and 30 mM Hepes. The baseline signal was collected for 2.5 min; then, cells were treated with 50 μM ML-SA1 or ML2-SA1 to activate the channel.

### Colocalization

To measure ER colocalization, HeLa cells were cotransfected with mCherry-tagged versions of TRPML plasmids and EGFP-KDEL (ER marker). To measure lysosomal colocalization, HeLa cells were transfected with mCherry-tagged TRPML plasmids. After 24 to 48 h, these cells were loaded with 1 μM LysoTracker Green (ThermoFisher). All cells were then washed and imaged in phosphate-free HHBSS buffer.

### Data analysis

Imaging data were analyzed with Fiji (ImageJ). Data output from Fiji was analyzed using both Excel and KaleidaGraph (version 5.01; Synergy Software). All experiments are representative of at least three independent experiments, and individual data points are technical replicates. Statistical analysis was performed using Excel or KaleidaGraph. One-way repeated-measures ANOVA with comparisons across all groups used post hoc Tukey's honest significant difference test, and comparisons with control used Dunnett’s test. All measurements were taken from distinct cytosolic regions of interest (ROIs) from each cell. Background fluorescence was subtracted from ROIs for time traces of fluorescence. The passive rate of permeability for a given ion was determined by measuring the peak ΔF/F_0_ value 2.5 min after treatment with the metal solution without agonist. The active rate of permeability was measured as the peak ΔF/F_0_ value at 1.5 min after the addition of the channel’s agonist. Colocalization analysis and determination of Pearson’s correlation coefficient (*R*) were performed using a custom ImageJ macro based on the Coloc two plugin (FIJI, NIH). For each image, contrast in both channels was linearly enhanced (0.35% saturation), and individual cells or ROIs were manually defined using the freehand selection tool. Following channel splitting, optional background subtraction (rolling ball radius = 5 pixels) was performed. The point spread function was set based on imaging conditions (60× 1.4 NA oil immersion objective and 1.5× OptiVar). Colocalization was then assessed as the Pearson’s correlation coefficient between channel 1 and channel 2 within each ROI using the Coloc 2 plugin.

### AlphaFold modeling and structural visualization

The structure of TRPML1 I468V was predicted using the AlphaFold server (Google DeepMind and Isomorphic Labs: https://alphafoldserver.com/, powered by AlphaFold 3) (56). The query consisted of the amino acid sequence of wildtype TRPML1 with a valine at residue 468 instead of an isoleucine. The prediction was run using standard settings, where five models were predicted, and corresponding predicted aligned error plots were produced. The structure of closed TRPML1 (Protein Data Bank code: 5WJ5) was overlaid with the top predicted structure of TRPML1 I468V predicted by AlphaFold. The final structure models do not include the first 40 residues, which are disordered and not modeled. Figure preparation and model visualization were performed using UCSF ChimeraX 1.10.

## Data availability

All data generated or analyzed during this study are included in this published article. All original imaging videos can be requested from the corresponding author.

## Supporting information

This article contains supporting information.

## Conflict of interest

The authors declare that they have no conflicts of interest with the contents of this article.
