# Supplementary material for: Differential ion selectivity and disease-associated dysfunction of TRPML channels revealed by patient and engineered mutants
Source: J Biol Chem. 2025 Nov 17;302(1):110953. doi: 10.1016/j.jbc.2025.110953 (PMC12753230; doi:10.1016/j.jbc.2025.110953)
Supplement: Supporting information [file mmc1.docx]

**Supporting Information**

**Figure S1**. TRPML1 is more permeable to Zn²⁺ compared to TRPML2 and TRPML3 at physiological conditions. A) Representative traces of agonist-mediated Zn²⁺ uptake (GZnP3 ∆F/F_0_) over time in HeLa cells overexpressing mCherry-tagged TRPML1, TRPML2, and TRPML3 on the plasma membrane (PM) co-transfected with GZnP3 in calcium-free buffer. Cells overexpressing TRPML1 and TRPML3 were incubated with 5 µM ZnCl_2_ and treated with 50 µM ML-SA1 to induce TRPML1/3 channel opening, whereas cells overexpressing TRPML2 were treated with 50 µM ML2-SA1. B) Quantification of agonist-mediated Zn²⁺ uptake through TRPML1-3 channels based on the peak ∆F/F_0_ values of GZnP3 following ML-SA1 or ML2-SA1 activation. Traces were normalized to the timepoint before agonist addition. Data are presented as mean ± SEM, and individual data points are technical replicates. N = 12, TRPML1; N = 10, TRPML2; N = 9, TRPML3. One-way ANOVA with Tukey HSD multiple comparison. *p < 0.05, n.s. not significant.

**Figure S2.** AlphaFold3 predicts the model of TRPML1 I468V with high confidence. A) Model of TRPML1 I468V colored according to pLDDT scores (local confidence). B) Overlay of the five predicted models of TRPML1 I468V. Residues 1-40 are disordered and not modeled. C) Overlay of the five predicted models of TRPML1 I468V, specifically showing the closed ion-conduction pore. Only two diagonal subunits are shown for clarity. The following pore-lining residues are shown as sticks: V468, N469, D471, D472.
